# Velocity distribution in active particles systems

**DOI:** 10.1038/srep23297

**Published:** 2016-03-22

**Authors:** Umberto Marini Bettolo Marconi, Nicoletta Gnan, Matteo Paoluzzi, Claudio Maggi, Roberto Di Leonardo

**Affiliations:** 1 Scuola di Scienze e Tecnologie, Università di Camerino, Via Madonna delle Carceri, 62032, Camerino, INFN Perugia, Italy; 2 CNR-ISC, UOS Sapienza, P.le A. Moro2, I-00185, Roma, Italy; 3 Physics Department, Syracuse University, Syracuse, NY, 13244, USA; 4 Dipartimento di Fisica, Università di Roma “Sapienza”, I-00185, Roma, Italy; 5 CNR-IMIP, UOS Roma, Dipartimento di Fisica Università Sapienza, I-00185, Roma, Italy

## Abstract

We derive an analytic expression for the distribution of velocities of multiple interacting active particles which we test by numerical simulations. In clear contrast with equilibrium we find that the velocities are coupled to positions. Our model shows that, even for two particles only, the individual velocities display a variance depending on the interparticle separation and the emergence of correlations between the velocities of the particles. When considering systems composed of many particles we find an analytic expression connecting the overall velocity variance to density, at the mean-field level, and to the pair distribution function valid in the limit of small noise correlation times. Finally we discuss the intriguing analogies and main differences between our effective free energy functional and the theoretical scenario proposed so far for phase-separating active particles.

The velocities of particles in any equilibrium classical system behave in a particularly simple way following the Maxwell-Boltzmann distribution^1^,^2^. However, when a system is driven out of equilibrium, the situation may change dramatically. In these systems we do not know generally what is the actual distribution of the velocities and strong deviations from the Maxwell-Boltzmann distribution may be observed. In granular systems, for example, non-Gaussian velocity distributions and cross correlations between the fluctuations of kinetic temperature and density have been found both in theoretical models^3^,^4^ and experiments^5^. The non-equilibrium behaviour of particle velocities is also a fundamental issue in a completely different type of far from equilibrium systems that is “active matter”^6^,^7^. This novel class of systems may be generally considered as composed by biological or synthetic “particles” that are capable of converting available energy into different kinds of persistent motion. This is the case for example of self-propelled bacteria such as *E. coli*, swimming along straight runs interrupted by random reorientations which can be modelled by the “run and tumble” (RT) dynamics^8^,^9^,^10^,^11^. Similarly, Janus-type colloids are propelled by chemical reactions and gradually reorient by rotational Brownian motion which is accounted for by the “active Brownian” (AB) model^12^,^13^,^14^,^15^,^16^. These systems show a ubiquitous tendency to accumulate near repulsive obstacles^17^,^18^,^19^ and often display clustering and/or phase separation even in the absence of attractive interactions^14^,^20^,^21^,^22^,^23^,^24^. In ref. 11 a very general mechanism accounting for these phenomena was proposed, inspired by the RT dynamics. The basic idea is that the velocities of the particles decrease rapidly where the local density increases and conversely the density becomes higher where the local velocity of the particles decreases. This feedback mechanism generates dense regions composed by slow particles and may eventually lead to the so-called “motility induced phase separation”. Although this elegant scenario was proposed some years ago several fundamental questions still remain unanswered: how do particles velocities depend on density? How do velocities depend on the interaction between particles and on the interparticle distance? To tackle these issues from a theoretical point of view, we consider the Gaussian colored noise (GCN) model^25^,^26^,^27^,^28^ which embodies the persistency of the active motion at the simplest level. Supplementing this model with the *multidimensional unified colored noise approximation* (MUCNA)^29^,^30^,^31^,^32^ we derive an explicit expression for the distribution of velocities of interacting active particles. We show that this distribution captures well the results of numerical simulation of the GCN model. This distribution shows explicitly that the velocities are coupled to positions via the Hessian matrix associated with the interaction potential. For two interacting particles only the individual velocities have a variance that depends on their distance. Moreover, the model predicts correlations between the velocities of the particles. For an active many-body system we derive an analytic expression connecting the overall velocity variance to the density by a mean-field approximation. We also find an expression connecting the velocity variance to the pair distribution function valid in the limit of small persistence time. We conclude by deriving an effective free energy functional and by comparing it to the one proposed in ref. 11 discussing the motility induced phase separation scenario for our model at the mean-field level.

## Results

### Main result

The GCN model is defined by the set of stochastic differential equations:


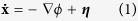


where the position variables 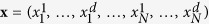, of the *N* particles in a *d*-dimensional space, are driven by the velocities −∇*ϕ* (generated by the conservative potential *ϕ*(**x**)). The vector ***η*** is a set of Ornstein-Uhlenbeck processes having 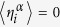 and 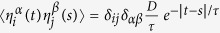 (where *i*, *j* = 1, …, *N* and 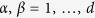). Here *D* is the diffusion coefficient of the particles in absence of interactions and *τ* is the relaxation time of the “propulsion” random forces. By using the MUCNA in ref. 25 we have found the approximated stationary probability for the GCN model:


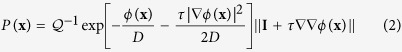


where 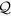 is a normalization factor obtained by integrating over **x**,**I** is the *dN* × *dN* identity matrix, ∇∇*ϕ* is the Hessian of *ϕ* and ||…|| represents the absolute value of the determinant of a matrix. The new and central result of the present work is the derivation, within the MUCNA, of the conditional probability 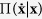 of having a velocity vector 
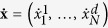
 given that the positions are *fixed* by the vector **x**:


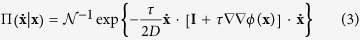


where 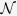 is a normalization factor, obtained by integrating over 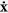, which depends parametrically on the **x**. To understand the basic principle of our derivation leading to Eq. (3) we briefly outline it here for a single degree of freedom. We rewrite Eq. (1) as the set of equations 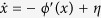 and 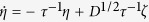 (where 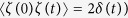. We proceed by further differentiating with respect to time the first equation and rewrite it as a second-order differential equation containing a white noise term : 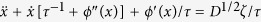. By using the scaled time *s* = *τ*^−1/2^*t* this equation becomes: 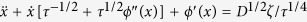. When the dynamics is cast in this form the key point of the UCNA becomes clear: the term 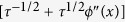 plays the role of a non-homogeneous friction and, if the potential curvature 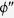 is positive, we can neglect the 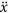 term (adiabatic approximation) both in the small and long *τ* limit (see ref. 29 for more details). Moreover from this second-order differential equation we can obtain a Kramers equation for the full phase-space distribution 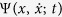:


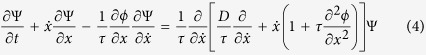


We now consider the stationary solution 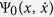. In this case, by multiplying Eq. (4) by 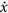 and integrating, we obtain:


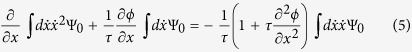


At this point we make the *antsatz* 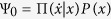, where Π has the Gaussian form of Eq. (3), and we substitute this into Eq. (5). With such a factorization Eq. (5) reduces to an ordinary differential equation for *P*(*x*), whose solution coincides precisely with the probability 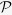 of Eq. (2) proving that Π is the correct velocity distribution within our approximation (see the Supplemental Material for details on the derivation for many degrees of freedom).

**Figure 1:**
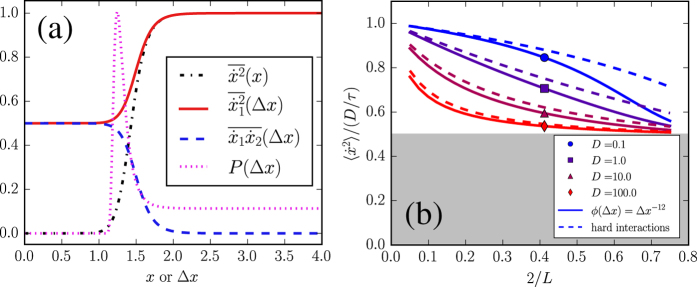
Velocity variance and correlation for two interacting particles. (**a**) Velocity variance and correlation as a function of distance for *D* = 1 and *τ* = 1 as computed from Eq. (3). The dashed-dotted line is the variance of velocity as a function of *x* for one particle moving in 1*d* in the presence of the repulsive barrier *ϕ* = *x*^−12^. The full line is the variance of velocity of one particle interacting with another particle via the repulsive potential *ϕ* = Δ*x*^−12^, the dashed line is the velocity correlation between the two interacting particles. The dotted line is the probability distribution given by Eq. (2) for the interacting particles and *L* = 8. (**b**) Overall velocity variance (Equation (6)) for two interacting active particles as a function of density for fixed *τ* = 1. Full lines with one symbol represent 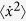 for the interaction *ϕ* = Δ*x*^−12^ at different values of *D* (see legend). The dashed line 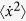 in the limiting case of two hard spheres. The shaded area represents the lower bound *D*/(2*τ*).

The result of Eq. (3) immediately tells us that the probability distribution of 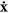 is a multivariate Gaussian but, very differently from the Maxwell-Boltzmann distribution, its covariance matrix depends on the positions via the Hessian. Let us start examining the implications of Eq. (3) by considering a single active particle moving in one dimension (1*d*) and subjected to an external potential. In this case Eq. (3) gives the variance as a function of 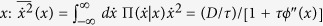 where the prime represents the derivative with respect to ***x***. From now on we will use the overbar to indicate specifically the averaging over the velocities: 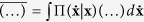, while the average over positions will be indicated by the brackets: 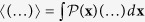. This shows that when the particle is in a region of high potential curvature its velocity variance decreases. To be more specific let us consider a purely repulsive potential of the form *ϕ* = *x*^−12^. The corresponding 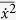 is plotted in Fig. 1(a) as a dashed-dotted line and this shows clearly that the velocity variance is close to the unperturbed value *D*/*τ* where the 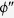 is small, but it decreases rapidly to zero in proximity to the repulsive “wall” generated by the external potential.

### Two interacting particles

We now consider two particles, with positions **x** = (*x*_1_, *x*_2_), interacting via the potential *ϕ*(*x*_1_−*x*_2_) = *ϕ*(Δ*x*) and moving in 1*d*. In this case Eq. (3) can be used to compute both the velocity variance: 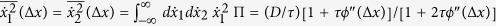 and the velocity correlations: 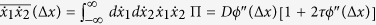. These equations show that the variance of the velocity decreases when the particles are at a distance where the interaction potential has high curvature and that in this situation also a correlation of velocities emerge. To visualize these quantities we consider the interaction potential 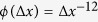 and we plot 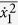 and 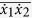 in Fig. 1(a) as a full and dashed line respectively. Since this potential has a curvature that goes rapidly to zero, 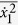 tends rapidly to the unperturbed value *D*/*τ*, while when the particles are close enough the value of 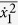 goes to *D*/(2*τ*). This limiting value represents the mean squared speed of the center of mass of the two particles system. Similarly, the correlation 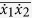 goes to zero at large Δ*x* (where interaction is small) while, when Δ*x* is small, the two particles will move coherently with the velocity of the center of mass resulting in the limiting value 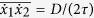.

Up to this point we have considered the velocity variance when the particles positions are fixed arbitrarily. However, we want also to compute this quantity averaging it over the positions to obtain the overall velocity variance of a GCN-driven system. This can be done, within the MUCNA, by combining Eqs (2) and (3):


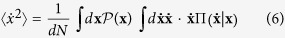


where, by dividing by *dN*, we average also over all particles and all components. To understand this result let us consider, as above, two particles in 1*d* interacting via *ϕ* = Δx^−12^ and assume that they move in a box of length *L* with periodic boundary conditions. The 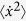 computed numerically via Eq. (6) is plotted in Fig. 1(b) (full lines) as a function of the 1*d* density *ρ* = 2/*L* of the system and for several values of *D* (at fixed *τ*). In Fig. 1(b) we divide the variance by the free-particle value *D*/*τ* so that 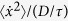 reduces to unity in absence of interactions. This shows clearly that 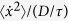 decreases systematically with increasing *ρ* and with increasing *D*. Upon increasing *D* we also observe a change in the convexity of the curves in Fig. 1(b). Such a phenomenology can be understood by noting that the 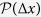 is very peaked around 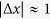 (see dotted line in Fig. 1(a)). Note also that the 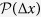 is practically zero when 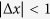 defining the “diameter” of the particles *σ* ≈ 1 that changes very little in the wide range of *D* and *τ* studied here. In ref. 25 we have shown that, by assimilating the potential *x*^−12^ to a hard potential, 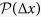 can be approximated by a Dirac delta of area 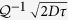 around the points Δ*x* ≈ ±σ and reduced to 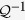 elsewhere, the normalization constant being 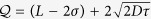. As discussed above in this limit we find also: 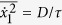 if 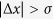, and 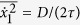 if 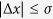. Combining these formulae we find the velocity variance of two GCN active hard spheres: 
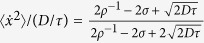
 which is a monotonic decreasing function of *ρ* and *D*. Moreover 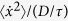 is bounded from below by 1/2 (in the limit of both large *ρ* and *D*). This is plotted as a dashed line in Fig. 1(b) and follows qualitatively well the results of the *ϕ* = Δ*x*^−12^ case. Moreover from this equation we can see that 
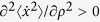
 if 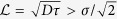 where we have introduced the characteristic length of the active motion 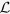. This, in practice, means that, the velocity variance becomes especially sensitive to density changes when the *Péclet number*^23^ *Pe* becomes of order one, i.e. 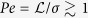.

### Many interacting particles

**Figure 2:**
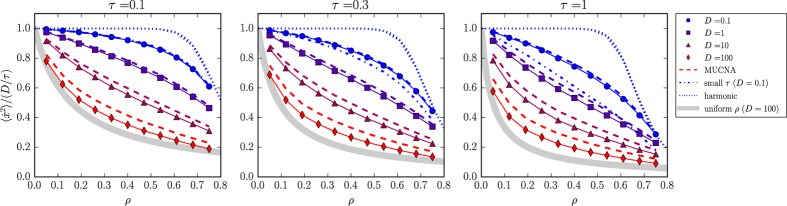
Normalized velocity variance for a 1*d* system of many interacting active particles. Symbols are the results of numerical simulations for several values of *τ* and *D* (see legend). Dashed lines are the theoretical velocity variances obtained by averaging Eq. (3) over the coordinates obtained numerically. Thick lines are the result of a homogenous density approximation. Dashed-dotted lines represent the small-*τ* approximation connecting the variance to the pair distribution function. Dotted lines are the velocity variances obtained by mapping the system onto a harmonic model.

To make progress towards the statistical description of the velocities of the many-body active system we consider *N* interacting particles in 1*d*. We perform numerical simulations of systems with *N* = 1000 composed by GCN-driven particles interacting via the potential 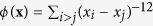 for several values of the density *ρ* = *N*/*L*, of *D* and *τ*. In all these simulations we compute the variance 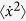 and report the results in Fig. 2 as connected symbols. The velocity variance is computed by averaging over all particle and over more than 3 × 10^4^ configurations. The relative error on this quantity can be estimated by computing its standard deviation over configurations and that results to be ≈5% (i.e. about the size of the symbols in Fig. 2). Qualitatively the emerging scenario seems to be close to the two-particle case (see Fig. 1(b)). However from a quantitative point of view the two-particle model is far from the results in Fig. 2, in particular the many-body 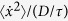 reaches values well below the lower bound of the two-particle case 1/2. To test uniquely the approximated distribution given by Eq. (3), we compute the average over positions in Eq. (6) directly from the coordinates obtained numerically, instead of using the theoretical 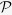 of Eq. (2). This is plotted in Fig. 2 as dashed lines and follows well the numerical curves, although some expected deviation^29^ is observed upon increasing *D* to very high values.

**Figure 3:**
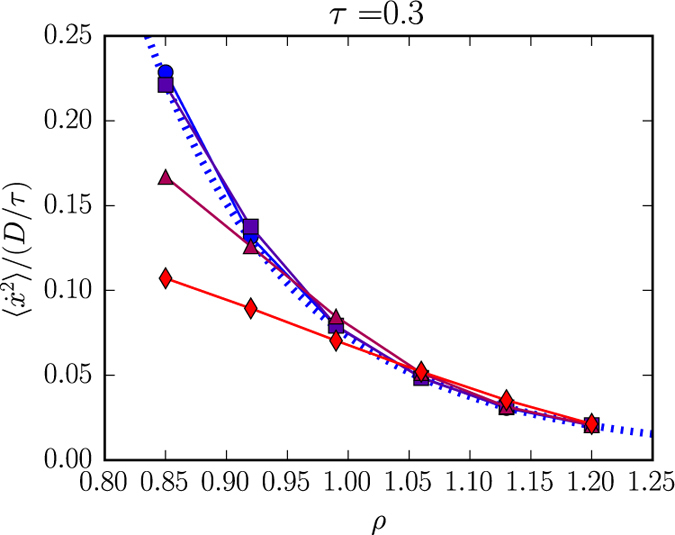
Harmonic model for the velocity variance. Comparison between the harmonic model (dotted line) and the numerical simulations (symbols) at high *ρ* for several values of *D* (same legend as Fig. 2) at fixed *τ* = 0.3.

If we assume a uniform density and long-ranged interactions (mean-field approximation) the velocity distribution (Eq. (3)) simplifies substantially. The terms on the diagonal of ∇∇*ϕ* take the form 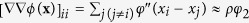, where *φ* is the pair potential and 
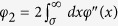
 is the mean potential curvature integrated up to the diameter *σ*. Similarly the out-of-diagonal terms of ∇∇*ϕ* become 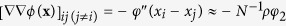. With this approximation the matrix **I** + τ∇∇*ϕ* has (*N* − 1) identical eigenvalues^33^ of the form 1 + (*N* + 1) *τφ*_2_*ρ*/*N*, while the *N*-th (non-degenerate) eigenvalue is 1 + *τφ*_2_*ρ*/*N*. The trace of the inverse matrix is therefore


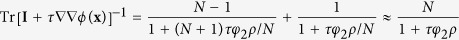


for large *N*. Dividing this result by *N* yields the density-dependent variance:


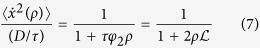


where in the last equality we have used the fact that, for a generic repulsive potential, *σ* corresponds roughly to the distance where the interaction force balances the self-propulsion (i.e. 
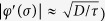
 and 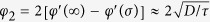. This is plotted in Fig. 2 as a thick line for the largest *D* and follows qualitatively well the data when 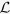 is large. When *τ* is small Eqs. (2) and (3) can be expanded to first order in *τ* (see Supplemental Material) which gives an alternative formula for *ϕ*_2_ in terms of the pair distribution function *g*(*x*), i.e. 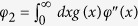. This is plotted in Fig. 2 as dashed-dotted lines and compares well with the numerical simulations at small values of *τ*. However, by fixing *τ* and increasing *D* this approximation deviates strongly from the simulations and therefore it is not shown in Fig. 2. In order to describe more accurately the high-density regime, we derive also a harmonic model for the velocity distribution. To this aim we consider a 1*d* system of active particles connected by springs having elastic constant *k*. In this system each particle is connected only to its nearest neighbour by a harmonic potential, the Hessian matrix in Eq. (3) does not depend on the positions anymore and it takes the form of a banded symmetric Toeplitz matrix whose elements are 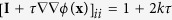, 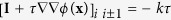 and zero else where. The eigenvalues of this matrix are known^34^ and the mean of their inverse can be computed as a Watson integral giving the result: 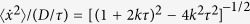. To compare this result with the simulations of the *ϕ* = Δ*x*^−12^ potential we assume that in the high *ρ* regime particles are separated by the average distance *ρ*^−1^ and expand the interaction potential to 2nd order around these distance. We obtain an estimate of the effective spring constant as *k*(*ρ*) = 156*ρ*^14^. The resulting 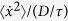 is plotted in Fig. 2 as a dotted line and it is found to well reproduce the numerical results at high values of *ρ* and *τ*, while some deviation is observed upon increasing *D*. However, by going at high densities (*ρ* ≈ 1) this harmonic model predicts quantitatively very well 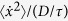 for all values of *D* and *τ* (see Fig. 3).

### Effective free energy functional

**Figure 4:**
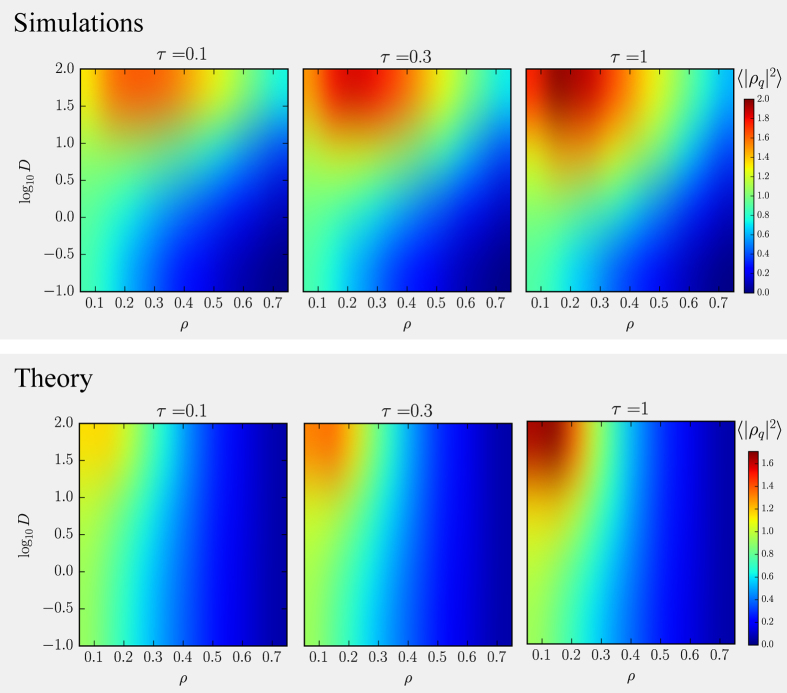
Density fluctuations for a many-body active system. (Top-panel) The colormap represents 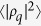 at low *q* obtained numerically for several values of *ρ*, *D* and *τ*. Colouring close to red indicates large density fluctuations at that state-point (*ρ*, *D*). (Bottom-panel) Theoretical value of 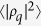 (computed from Equation (9)) shows a qualitative behaviour very similar to the one found in simulations.

We have seen that the probability distribution given by Eq. (2) can be mapped onto a Boltzmann distribution characterized by the effective potential 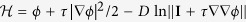. Exploiting this analogy, as we have shown in ref. 26, we can construct an effective free energy functional of the form 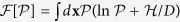. Moreover by using Eq. (3) we can rewrite 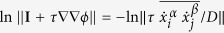 since the determinant of the inverse is the inverse of the determinant. Using these results we can recast 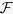 as


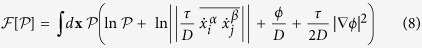


which shows a dependence on the velocities resembling closely the one suggested in ref. 11. In particular the term 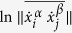 represents the differential entropy of the velocity distribution, i.e. how much velocities are “spread” in the velocity space. This implies that if velocities have some very high values, for a given configuration **x**, this brings positive contribution to free energy and therefore 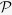 will be low for such **x**. Conversely, if velocities concentrate around zero at **x**, 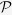 will grow for this configuration. Note also that we know how to express the velocity term in Eq. (8) as a function of density, at least at the mean field level in 1*d*, by using Eq. (7). This further suggests that the velocity variance is itself a decreasing function of density establishing the feedback mechanism hypothesized in ref. 11. Moreover, if we assume a homogeneous *ρ* and only repulsive interactions, the terms *ϕ* and |∇*ϕ*|^2^ correspond only to repulsive potential terms in Eq. (8). With these considerations we rewrite Eq. (8) in the mean-field form:


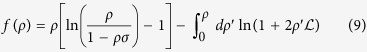


where we have introduced the free energy per unit length 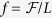 and we have absorbed all the repulsive terms in the 1*d* hard-spheres excess free energy −*ρ* ln (1−*ρσ*). If 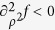 the homogeneous density phase is unstable and the system undergoes a spinodal decomposition. In contrast to this scenario, we find from the mean-field Eq. (9) always gives 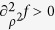. This suggests that our active system does not phase separate for any value of *ρ*, *D* and *τ* even in the presence of long-ranged interactions. This is consistent with the numerical results that do not show any discontinuity in any of the average values that we have monitored. However Eq. (9) does predict an anomalous behaviour of density fluctuations. To show this we consider the 1*d* Fourier transformed density fluctuations 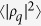, where 
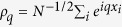
 (*q* being the wavevector). We study the long wave-length density fluctuations by choosing *q* ≈ (20*σ*)^−1^ and compute 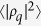 in numerical simulations which is plotted in Fig. 4 (top panel) as a colormap for several values of *D*, *ρ* and *τ*. This shows that at sufficiently high *D* the 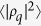 develops a maximum as a function of the average density, *ρ* and that this maximum increases in amplitude as *τ* increases. At such small *q* we can approximate^35^ 
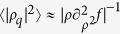
 which is plotted in Fig. 4 (bottom panel). This is found to follow qualitatively very well the numerical results in the whole *D*, *τ* and *ρ* range explored. In practice, the effect of the colored noise is to introduce a sort of weak effective attraction at high persistence lengths 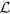 enhancing the density fluctuations in the low-density regime with respect to a purely repulsive equilibrium system. In this low-*ρ*/high-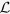 regime an evident clustering of the particles is generated by the persistent propulsion forces.

## Discussion

By using the unified colored noise approximation, we have derived an explicit expression for the distribution of velocities of interacting active particles and shown that it agrees well with the numerical simulation of the GCN model. This distribution predicts the non-equilibrium coupling between velocities and positions and the correlation between velocities of different particles. These have been characterized in detail for the two-particle case. In the case of many interacting active particles, we have derived approximations connecting the velocity variance to the pair distribution function at low values of the persistence time. From our microscopic model, we have also derived directly an effective free energy functional that shows surprising similarities with the one proposed so far for describing the phase separation in active particles. Our functional establishes directly a connection between the stationary probability of coordinates and the covariance matrix of velocities showing that a configuration with low velocities is favoured. Moreover, our theory shows that when interactions are strong (i.e. when density is high) the velocities tend to decrease generating a feedback mechanism that enhances density fluctuations. The basic physics of this phenomenon is captured by a mean-field version of our functional that predicts qualitatively well the excess density fluctuations observed numerically. These anomalous density fluctuations indicate clustering of the particles in analogy with the cluster formation observed experimentally for Janus colloids^14^. However, in the present one-dimensional mean-field model, this mechanism is too weak to lead to spinodal decomposition. Simulation results (see Supplemental Material) show that this scenario is also found in two-dimensional systems driven by GCN. The velocity variance obtained in 2d is still a decreasing function of density although an interesting non-monotonic behavior as a function of the diffusivity is found. Moreover anomalous density fluctuations and clustering (without phase separation) are found also in 2d. As expected the qualitative mean-field picture is not changed by further increasing the dimensionality. This marks a clear difference between our model and the AB model which has been shown to phase separate. It has been noted that, in the GCN model, the magnitude of the propulsive forces can fluctuate^36^,^37^ and occasionally reach very high values and that could destabilize the condensed phase. Differently this mechanism should not be present in AB systems where the propulsion force is always bounded. Moreover it has been shown^28^ that the AB model and the GCN model can display quite different stationary distributions in presence of external potentials. In that particular case it has been found that noise distributions having different shapes can lead to rather different stationary density profiles. Interestingly however it was shown in ref. 22 that a further approximation of the GCN theoretical framework could predict the phase separation in AB particles. With this perspective it would be interesting to extend our mean-field model to the3*d* case, accounting explicitly for the potential terms, and perform exhaustive 3*d* simulations of the GCN model.

## Additional Information

**How to cite this article**: Marconi, U. M. B. *et al.* Velocity distribution in active particles systems. *Sci. Rep.* **6**, 23297; doi: 10.1038/srep23297 (2016).

## Supplementary Material

Supplementary Information
