# Corrigendum: Velocity distribution in active particles systems

**DOI:** 10.1038/srep26215

**Published:** 2016-05-31

**Authors:** Umberto Marini Bettolo Marconi, Nicoletta Gnan, Matteo Paoluzzi, Claudio Maggi, Roberto Di Leonardo

Scientific Reports 6: Article number: 2329710.1038/srep23297; published online: 03222016; updated: 05312016

In this Article, Roberto Di Leonardo is incorrectly affiliated with ‘CNR-IMIP, UOS Roma, Dipartimento di Fisica Università Sapienza, I-00185, Roma, Italy’. The correct affiliation is listed below:

NANOTEC-CNR, Institute of Nanotechnology, Soft and Living Matter Laboratory, Roma, Italy.

