# Supplementary material for: Velocity distribution in active particles systems
Source: Sci Rep. 2016 Mar 22;6:23297. doi: 10.1038/srep23297 (PMC4802341; doi:10.1038/srep23297)
Supplement: Supplementary Information [file srep23297-s1.pdf]

Velocity distribution in active particles systems, Supplemental Material

Umberto Marini Bettolo Marconi,[1] Claudio Maggi,[2] Nicoletta Gnan,[2] Matteo Paoluzzi,[3] and Roberto Di Leonardo[2]

[1] *Scuola di Scienze e Tecnologie, Università di Camerino, Via Madonna delle Carceri, 62032, Camerino, INFN Perugia, Italy*

[2] *Universita Sapienza, Rome, Italy*

[3] *Syracuse University, NY, USA*

(Dated: February 19, 2016)

A simple derivation of the stationary velocity distribution of a collection of active particles subject to non equilibrium colored noise is provided in this note. Moreover simulation results for the 2-dimensional case are reported.

I. DERIVATION OF THE STATIONARY VELOCITY DISTRIBUTION

The effective dynamics for space coordinates of an assembly of active spheres[1,2] is

$$\dot{\mathbf{r}}_i(t) = \frac{1}{\gamma}\boldsymbol{F}_i(\mathbf{r}_1, \dots, \mathbf{r}_N) + \boldsymbol{u}_i(t) \tag{1}$$

where the velocities $\boldsymbol{u}_i$ evolve according to the law:

$$\dot{\boldsymbol{u}}_i(t) = -\frac{1}{\tau}\boldsymbol{u}_i(t) + \frac{D_a^{1/2}}{\tau}\boldsymbol{\eta}_i(t) \tag{2}$$

The force $\boldsymbol{F}_i = -\nabla_i \mathcal{U}$ acting on the i-th particle is conservative and associated to the potential $\mathcal{U}(\mathbf{r}_1, \dots, \mathbf{r}_N)$, γ is the drag coefficient, whereas the stochastic vectors $\eta_i(t)$ are Gaussian and Markovian processes distributed with zero mean and moments $\langle \boldsymbol{\eta}_i(t)\boldsymbol{\eta}_j(t')\rangle = 2\delta_{ij}\delta(t-t')$. where d is the spatial dimensionality. The coefficient D_a due to the activity is related to the correlation of the Ornstein-Uhlenbeck process $\boldsymbol{u}_i(t)$ via

$$\langle \boldsymbol{u}_i(t)\boldsymbol{u}_j(t')\rangle = d\frac{D_a}{\tau}\delta_{ij}\exp(-|t-t'|/\tau).$$

where d is the spatial dimension. To simplify the notation we switch from $\mathbf{r}_i$ to an array x_i as done in previous publications:

$$\dot{x}_i(t) = \frac{1}{\gamma}F_i(x_1, \dots, x_N) + u_i(t) \tag{3}$$

Differentiate again

$$\ddot{x}_i(t) = \frac{1}{\gamma}\sum_k \frac{\partial F_i}{\partial x_k}\dot{x}_k + \dot{u}_i(t) \tag{4}$$

$$\ddot{x}_i(t) = \frac{1}{\gamma}\sum_k \frac{\partial F_i}{\partial x_k}\dot{x}_k - \frac{1}{\tau}[\dot{x}_i - \frac{F_i}{\gamma}] + \frac{D_a^{1/2}}{\tau}\eta_i \tag{5}$$

Let us introduce the variable v_i and recast eq.(5) as:

$$\dot{x}_i = v_i \tag{6}$$

$$\dot{v}_i = \frac{1}{\gamma}\sum_k \frac{\partial F_i}{\partial x_k}v_k - \frac{1}{\tau}[v_i - \frac{F_i}{\gamma}] + \frac{D_a^{1/2}}{\tau}\eta_i \tag{7}$$

A. One particle

Before presenting the multidimensional result, we digress to illustrate the kinetic method of solution in a simple one-dimensional case. We begin with a single particle in one dimension and drop the index i. We differentiate eq. (1) with respect to time and introduce the velocity variable $v = \dot{x}$ so that instead of the original system (1) and (2) we have:

$$\begin{aligned} \dot{v} &= -\frac{1}{\tau}(1 - \frac{\tau}{\gamma}\frac{dF}{dx})v + \frac{1}{\tau\gamma}F + \frac{D_a^{1/2}}{\tau}\eta \\ \dot{x} &= v \end{aligned} \tag{8}$$

We obtain the Kramers equation for the phase-space distribution $f(x, \dot{x}; t)$:

$$\frac{\partial f}{\partial t} + v\frac{\partial f}{\partial x} - \frac{\nabla \mathcal{U}}{\gamma\tau}\frac{\partial f}{\partial v} = \frac{1}{\tau}\frac{\partial}{\partial v}\Big(\frac{D}{\tau}\frac{\partial}{\partial v} + \Gamma(x)v\Big)f \tag{9}$$

with $\Gamma(x) = 1 + \frac{\tau}{\gamma}\nabla^2\mathcal{U}(x))$. By multiplying and integrating over v eq. (9) and considering only time independent solutions $f_0(x, v)$ one obtains:

$$\frac{\partial}{\partial x}\int dv v^2 f_0(x, v) + \frac{\nabla \mathcal{U}}{\gamma\tau}\int dv f_0(x, v) = -\frac{1}{\tau}\Gamma(x)\int dv v f_0(x, v) \tag{10}$$

Such an integro-differential equation can be solved by the following ansatz $f_0(x, v) = \Pi(v|x))\,\mathcal{P}(x)$ where Π is the Gaussian velocity distribution : $\Pi(v|x) = (\frac{\tau}{2\pi D})^{1/2}\sqrt{\Gamma(x)}\,\exp\Big(-\frac{\tau}{2D}\Gamma(x)v^2\Big)$ whose width depends on the particle position and the average velocity $\overline{v}$ vanishes. After substituting the factorization f_0 into (9) and evaluating the velocity variance

$$\int dv v^2\,\Pi(v|(x) = \frac{D_a}{\tau}\frac{1}{\Gamma(x)} \tag{11}$$

we arrive at the following differential equation determining the steady state coordinate distribution $\mathcal{P}(x)$:

$$\frac{d}{dx}[\Gamma^{-1}(x)\mathcal{P}(x)] + \frac{\nabla\mathcal{U}(x)}{D\gamma}\mathcal{P}(x) = 0 \tag{12}$$

which is identical to the differential equation determining the stationary coordinate distribution in the unified color noise approximation (UCNA):

$$\mathcal{P}(x) = \frac{1}{Z_1}\,\exp(\frac{-\mathcal{H}(x)}{T_s}) \tag{13}$$

with

$$\mathcal{H}(x) = \mathcal{U}(x) + \frac{\tau}{2\gamma}(\frac{\partial\mathcal{U}(x)}{\partial x})^2 - T_s\ln[1 + \frac{\tau}{\gamma}\frac{d^2\mathcal{U}(x)}{dx^2}] \tag{14}$$

and $T_s = D_a\gamma$ and Z_1 a normalization constant defined as:

$$Z_1 = \int dx\,\exp\Big(-\frac{\mathcal{H}(x)}{T_s}\Big). \tag{15}$$

The method can be easily extended to the multidimensional case.

II. A KINETIC DERIVATION OF THE STEADY DISTRIBUTION, VELOCITY CORRELATIONS FOR MANY PARTICLE SYSTEMS

We now generalize the kinetic argument above illustrated to a many particle system and write the following multi-dimensional Kramers equation[3] describing the evolution of the phase-space distribution $f_N(x_1, \ldots, x_N; , v_1 \ldots, v_N; t)$

$$\frac{\partial f_N}{\partial t} + \sum_i v_i\frac{\partial f_N}{\partial x_i} + \sum_i \frac{F_i}{\gamma\tau}\frac{\partial f_N}{\partial v_i} = \frac{1}{\tau}\sum_i\frac{\partial}{\partial v_i}\Big(\frac{D_a}{\tau}\frac{\partial}{\partial v_i} + \sum_k \Gamma_{ik}v_k\Big)f_N \tag{16}$$

with the non dimensional friction matrix Γ_{ik} defined as

$$\Gamma_{ik} = \delta_{ik} + \frac{\tau}{\gamma}\frac{\partial^2 \mathcal{U}}{\partial x_i \partial x_k}. \tag{17}$$

The probability conservation and the momentum balance equations are straightforwardly obtained by projection, i.e. by integrating eq.(16) over the dN dimensional velocity space after multiplying by 1 and v_i, respectively:

$$\frac{\partial P_N(\{x_i\},t)}{\partial t} + \sum_i \frac{\partial J_i(\{x_i\},t)}{\partial x_i} = 0 \tag{18}$$

$$\frac{\partial J_i(\{x_i\},t)}{\partial t} + \sum_k \frac{\partial p_{ik}(\{x_i\},t)}{\partial x_k} - \frac{F_i(\{x_i\},t)}{\gamma\tau} P_N(\{x_i\},t) = -\frac{1}{\tau}\sum_k \Gamma_{ik}(\{x_i\},t) J_k(\{x_i\},t) \tag{19}$$

where $P_N(\{x_i\},t)$ is the marginalized distribution giving the distribution of positions of the particles regardless their velocities:

$$P_N(\{x_i\},t) = \int d^N v\, f_N(\{x_i\},(\{v_i\},t)\,, \tag{20}$$

while the current $J_i(\{x_i\},t)$ is the N-dimensional vector:

$$J_i(\{x_i\},t) = \int d^N v\, v_i f_N(\{x_i\},(\{v_i\},t). \tag{21}$$

Finally, $p_{ij}(\{x_i\},t)$ is the $N \times N$ dimensional generalized kinetic pressure tensor

$$p_{ij}(\{x_i\},t) = \int d^N v\, v_i v_j f_N(\{x_i\},(\{v_i\},t) \tag{22}$$

Equations (18)-(19) are an exact consequence of (16), but require the knowledge of p_{ij} which can be obtained by continuing the projection procedure in velocity space to higher order in v_i and closing the hierarchy by a suitable truncation ansatz, such as the introduction of phenomenological transport coefficients. On the other hand, if we limit ourselves to study the stationary regime the approach is fruitful and leads to a different interpretation of the MUCNA equations. Let us first, notice that the operator featuring in the right hand side of (16) posseses a null eigenvalue whose associated eigenfunction (the ground state) is the multivariate Gaussian velocity distribution :

$$\Pi((\{v_i\}|\{x_i\}) = (\frac{\tau}{2\pi D_a})^{N/2}\sqrt{\det\Gamma}\, \exp\Big(-\frac{\tau}{2D_a}\sum_{ij} v_i \Gamma_{ij}(\{x\}) v_j\Big) \tag{23}$$

In other words $\Pi(\{v_i\}|\{x_i\})$ is the conditional probability distribution of velocities given the positions take on the values $\{x_i\}$. We now construct a time-independent trial phase-space distribution having a factorized form:

$$f_T^{(K)}(\{x_i\},(\{v_i\}) = \Pi((\{v_i\}|\{x_i\}) \times P^{st}(\{x_i\}) \tag{24}$$

and corresponding to zero current $J_i(x_1,\dots,x_N)$. For generic potentials the velocity moments of G are non constant in space, but depend on coordinates x_i and different velocity components may be correlated and are given by the formula:

$$\overline{v_i v_j} = \int d^N v v_i v_j \Pi(\{v_i\}|\{x_i\}) = \frac{D_a}{\tau}\Gamma^{-1}_{ij}(\{x_i\}) \tag{25}$$

so that the "pressure " tensor reads $p_{ij}(\{x_i\}) = \frac{D_a}{\tau}\Gamma^{-1}_{ij}(\{x_i\})P^{st}(\{x_i\})$. Using eq. (19) in conjunction with the condition of vanishing currents, $J_i = 0$, we have the following balance equations:

$$\frac{\tau}{D_a}\sum_k \frac{\partial p_{ik}(\{x_i\})}{\partial x_k} - \frac{F_i(\{x_i\})}{D_a\gamma}P^{st}(\{x_i\}) = 0 \tag{26}$$

Eliminating the pressure tensor using (25) we arrive at the differential equation determining $P^{st}(\{x_i\})$:

$$\sum_k \frac{\partial}{\partial x_k}[\Gamma^{-1}_{ik}(\{x_i\})P^{st}(\{x_i\})] + \frac{1}{D_a\gamma}\frac{\partial \mathcal{U}(\{x_i\})}{\partial x_i}P^{st}(\{x_i\}) = 0 \tag{27}$$

Finally, by multiplying by the matrix Γ equation (27) we arrive at an equation identical to the MUCNA equation (see ref[2])

$$-(D_a+D_t)\gamma\Big(\frac{\partial P_N}{\partial x_n}-P_N\frac{\partial}{\partial x_n}\ln\det\Gamma\Big)-P_N\sum_k\Big(\delta_{nk}+\frac{\tau}{\gamma}\frac{\partial^2\mathcal{U}}{\partial x_n\partial x_k}\Big)\frac{\partial\mathcal{U}(x_1,\dots,x_N)}{\partial x_k}=0. \tag{28}$$

in the case $D_t=0$, so that we may identify the configurational part of the trial solution P^{st} with P_N derived by the MUCNA method[2]:

$$P_N(x_1,\dots,x_N)=\frac{1}{Z_N}\exp\Big\{-\frac{1}{D_a\gamma}\Big[\mathcal{U}(x_1,\dots,x_N)+\frac{\tau}{2\gamma}\sum_k^N\Big(\frac{\partial\mathcal{U}(x_1,\dots,x_N)}{\partial x_k}\Big)^2-D_a\gamma\ln|\det\Gamma_{ik}|\Big]\Big\} \tag{29}$$

where Z_N is a normalization constant.

We turn now to the formula for the velocity covariance matrix for many particles. Using the Gaussianity of the distribution we can immediately write the averages of the velocity as

$$< v_i v_j >=\int d^N x\,\overline{v_i v_j}P_N(\{x_i\})=\frac{D_a}{\tau}\int d^N x\Gamma^{-1}_{ij}(\{x_i\})P_N(\{x_i\}) \tag{30}$$

where $\langle\cdot\rangle$ stands for the double average over velocities and over space since the velocity distribution depends on the positions, unlike the equilibrium case.

In the general case we must evaluate the matrix elements Γ_{ij}. As shown in the appendix this can be done to first order in τ/γ with the result for the velocity self-correlations:

$$< v^i_\alpha v^i_\alpha >=\frac{D_a}{\tau}\int d^N\mathbf{r}P^{st}(\mathbf{r}_1,\dots,\mathbf{r}_N)[1-\frac{\tau}{\gamma}\sum_{j\neq i}w_{\alpha\alpha}(\mathbf{r}_i,\mathbf{r}_j)] \tag{31}$$

Finally, averaging over the whole system and introducing the positional pair correlation function $g(\mathbf{r}-\mathbf{r}')$ one obtains

$$\frac{1}{N}\sum_{i=1}^N< v^i_\alpha v^i_\alpha >=\frac{D_a}{\tau}\Big(1-\frac{\tau}{\gamma}\rho\int d\mathbf{r}g_2(\mathbf{r})w_{\alpha\alpha}(\mathbf{r})\Big) \tag{32}$$

In analogy with granular gases we can define the second moment of the velocity distribution to be the average kinetic temperature, T^k of the system, via $T_k=\frac{1}{N}\sum_i^N\langle v_i v_i\rangle$.

III. APPROXIMATION FOR THE DETERMINANT AND VELOCITY CORRELATIONS

The exact evaluation of the determinant Γ associated with the Hessian matrix is beyond the authors capabilities and we look for approximations in order to evaluate the effective forces. We consider the associated determinant in the case of two spatial dimensions and vanishing external potential:

$$\begin{pmatrix}
[1+\frac{\tau}{\gamma}\sum_{j\neq1}w_{xx}(\mathbf{r}_1,\mathbf{r}_j)] & \sum_{j\neq1}\frac{\tau}{\gamma}w_{xy}(\mathbf{r}_1,\mathbf{r}_j) & -\frac{\tau}{\gamma}w_{xx}(\mathbf{r}_1,\mathbf{r}_2) & \dots & -\frac{\tau}{\gamma}w_{xy}(\mathbf{r}_1,\mathbf{r}_N)\\
\sum_{j\neq1}\frac{\tau}{\gamma}w_{yx}(\mathbf{r}_1,\mathbf{r}_j) & [1+\frac{\tau}{\gamma}\sum_{j\neq1}w_{yy}(\mathbf{r}_1,\mathbf{r}_j)] & -\frac{\tau}{\gamma}w_{yx}(\mathbf{r}_1,\mathbf{r}_2) & \dots & -\frac{\tau}{\gamma}w_{yy}(\mathbf{r}_1,\mathbf{r}_N)\\
-\frac{\tau}{\gamma}w_{xx}(\mathbf{r}_2,\mathbf{r}_1) & -\frac{\tau}{\gamma}w_{yx}(\mathbf{r}_2,\mathbf{r}_1) & [1+\frac{\tau}{\gamma}\sum_{j\neq2}w_{xx}(\mathbf{r}_2,\mathbf{r}_j)] & \dots & -\frac{\tau}{\gamma}w_{xy}(\mathbf{r}_2,\mathbf{r}_N)\\
\dots & \dots & \dots & \dots & \dots\\
-\frac{\tau}{\gamma}w_{xy}(\mathbf{r}_N,\mathbf{r}_1) & -\frac{\tau}{\gamma}w_{yy}(\mathbf{r}_N,\mathbf{r}_1) & \dots & \dots & [1+\frac{\tau}{\gamma}\sum_{j\neq N}w_{yy}(\mathbf{r}_N,\mathbf{r}_j)]
\end{pmatrix}$$

It is interesting to remark that the off-diagonal elements contain only one term, while the diagonal elements and their neighbors contain N terms. Thus in the limit of $N\to\infty$ we expect that the matrix becomes effectively diagonal. However, even with such a limit, we see that to order τ/γ the inverse matrix, which is directly related to velocity

correlator $\overline{v_{\alpha i}v_{\beta j}}$ (see (25)), reads:

$$\frac{\tau}{D}\overline{v_{\alpha i}v_{\beta j}} \approx \begin{pmatrix} [1-\frac{\tau}{\gamma}\sum_{j\neq 1} w_{xx}(\mathbf{r}_1,\mathbf{r}_j)] & -\sum_{j\neq 1}\frac{\tau}{\gamma}w_{xy}(\mathbf{r}_1,\mathbf{r}_j) & \frac{\tau}{\gamma}w_{xx}(\mathbf{r}_1,\mathbf{r}_2) & \dots & \frac{\tau}{\gamma}w_{xy}(\mathbf{r}_1,\mathbf{r}_N) \\ -\sum_{j\neq 1}\frac{\tau}{\gamma}w_{yx}(\mathbf{r}_1,\mathbf{r}_j) & [1-\frac{\tau}{\gamma}\sum_{j\neq 1} w_{yy}(\mathbf{r}_1,\mathbf{r}_j)] & \frac{\tau}{\gamma}w_{yx}(\mathbf{r}_1,\mathbf{r}_2) & \dots & \frac{\tau}{\gamma}w_{yy}(\mathbf{r}_1,\mathbf{r}_N) \\ \frac{\tau}{\gamma}w_{xx}(\mathbf{r}_2,\mathbf{r}_1) & \frac{\tau}{\gamma}w_{yx}(\mathbf{r}_2,\mathbf{r}_1) & [1-\frac{\tau}{\gamma}\sum_{j\neq 2} w_{xx}(\mathbf{r}_2,\mathbf{r}_j)] & \dots & \frac{\tau}{\gamma}w_{xy}(\mathbf{r}_2,\mathbf{r}_N) \\ \dots & \dots & \dots & \dots & \dots \\ \frac{\tau}{\gamma}w_{xy}(\mathbf{r}_N,\mathbf{r}_1) & \frac{\tau}{\gamma}w_{yy}(\mathbf{r}_N,\mathbf{r}_1) & \dots & \dots & [1-\frac{\tau}{\gamma}\sum_{j\neq N} w_{yy}(\mathbf{r}_N,\mathbf{r}_j)] \end{pmatrix}$$

We, now, assume translational invariance and perform the average over the spatial distribution $P_N(\mathbf{r}_1,\dots,\mathbf{r}_N)$, and introduce the pair correlation as $P_2(\mathbf{r}_1,\mathbf{r}_2)=\frac{\rho^2}{N(N-1)}g(\mathbf{r}_1-\mathbf{r}_2)$. The sought average is:

$$\frac{\tau}{D} < v_{\alpha i}v_{\beta j} >= \begin{pmatrix} 1-\frac{\tau}{\gamma}\rho\int d\mathbf{r}w_{xx}(r)g(r) & -\frac{\tau}{\gamma}\rho\int d\mathbf{r}w_{xy}(r)g(r) & \frac{\tau}{N\gamma}\rho\int d\mathbf{r}w_{xx}(r)g(r) & \dots & \frac{\tau}{N\gamma}\rho\int d\mathbf{r}w_{xy}(r)g(r) \\ -\rho\frac{\tau}{\gamma}\int d\mathbf{r}w_{yx}(r)g(r) & 1-\frac{\tau}{\gamma}\rho\int d\mathbf{r}w_{yy}(r)g(r) & \frac{\tau}{N\gamma}\rho\int d\mathbf{r}w_{yx}(r) & \dots & \frac{\tau}{N\gamma}\rho\int d\mathbf{r}w_{yy}(r)g(r) \\ \frac{\tau}{N\gamma}\rho\int \mathbf{r}w_{xx}(r)g(r) & \frac{\tau}{N\gamma}\rho\int d\mathbf{r}w_{yx}(r)g(r) & 1-\frac{\tau}{\gamma}\rho\int d\mathbf{r}w_{xx}(r)g(r) & \dots & \frac{\tau}{N\gamma}\rho\int d\mathbf{r}w_{xy}(r)g(r) \\ \dots & \dots & \dots & \dots & \dots \\ \dots & \dots & \dots & \dots & \dots \end{pmatrix}$$

One can see that in the limit of large N the off-diagonal terms become neglegible because they carry factors $1/N$ and one obtains a block diagonal matrix whose blocks are 2×2 matrices:

$$\begin{pmatrix} < v_x v_x > & < v_x v_y > \\ < v_y v_x > & < v_y v_y > \end{pmatrix} = \frac{D}{\tau}\begin{pmatrix} 1-\frac{\tau}{\gamma}\rho\int d\mathbf{r}w_{xx}(r)g(r) & -\frac{\tau}{\gamma}\rho\int d\mathbf{r}w_{xy}(r)g(r) \\ -\rho\frac{\tau}{\gamma}\int d\mathbf{r}w_{yx}(r)g(r) & 1-\frac{\tau}{\gamma}\rho\int d\mathbf{r}w_{yy}(r)g(r) \end{pmatrix}$$

If the system is rotationally invariant $< v_x v_x >=< v_y v_y >$ and $< v_x v_y >=< v_y v_x >= 0$.

A. Evaluation of the ensemble average of the determinant to linear order in τ/γ and velocity variance

Notice that to linear order τ/γ the ensemble average of the determinant is:

$$\int d\mathbf{r}_1\dots d\mathbf{r}_N P_N(\mathbf{r}_1,\dots,\mathbf{r}_N)\det\Gamma(\mathbf{r}_1,\dots,\mathbf{r}_N) \approx \Big([1+\frac{\tau}{\gamma}\rho\int d\mathbf{r}w_{xx}(r)g(r)][1+\frac{\tau}{\gamma}\rho\int d\mathbf{r}w_{yy}(r)g(r)]\Big)^N \tag{33}$$

$$\int d\mathbf{r}_1\dots d\mathbf{r}_N P_N(\mathbf{r}_1,\dots,\mathbf{r}_N)\det\Gamma(\mathbf{r}_1,\dots,\mathbf{r}_N) \approx \Big(1+\frac{\tau}{\gamma}\rho\int d\mathbf{r}[w_{xx}(r)+w_{yy})(r)]g(r)\Big)^N \tag{34}$$

To order τ/γ:

$$\int d\mathbf{r}_1\dots d\mathbf{r}_N P_N(\mathbf{r}_1,\dots,\mathbf{r}_N)\ln\det\Gamma \approx N\ln\Big(1+\frac{\tau}{\gamma}\rho\int d\mathbf{r}[w_{xx}(r)+w_{yy}(r)]g(r)\Big) \approx N\ln\Big(\frac{D}{\tau}\frac{1}{< v_x v_x >}\Big)+N\ln\Big(\frac{D}{\tau}\frac{1}{< v_y v_y >}\Big) \tag{35}$$

IV. 2D SIMULATION RESULTS

We simulate a $2d$ system driven by GCN and composed by $N=1000$ particles with periodic boundary conditions. The particles interact via the purely repulsive pair potential $\varphi(\mathbf{r}_1-\mathbf{r}_2)=|\mathbf{r}_1-\mathbf{r}_2|^{-12}$. We fix $\tau=1$ and we explore several values of density $\rho=N/L^2$ and diffusivity D. The results are reported in Fig. 1. Fig. 1(a) shows the normalized velocity variance as a function of density for different values of D. It is seen that, as in the $1d$ case, the quantity $\langle\dot{x}^2\rangle/(D/\tau)$ is a decreasing function of ρ. However in the $1d$ case $\langle\dot{x}^2\rangle/(D/\tau)$ is also a decreasing function of D (at fixed ρ), while in $2d$ a non-monotonic behavior in D can be observed at high ρ. Fig. 1(b) shows the amplitude of the Fourier-transformed density fluctuations at low q ($q\approx(20\sigma)^{-1}$) which shows a very similar behaviour to the $1d$ case. Fig. 1(c) and (d) show two snapshots at high D and intermediate densities where evident clustering of the particles is observed but not a full phase separation. This is the case also upon further increasing τ to very high values as shown in Fig. 2.

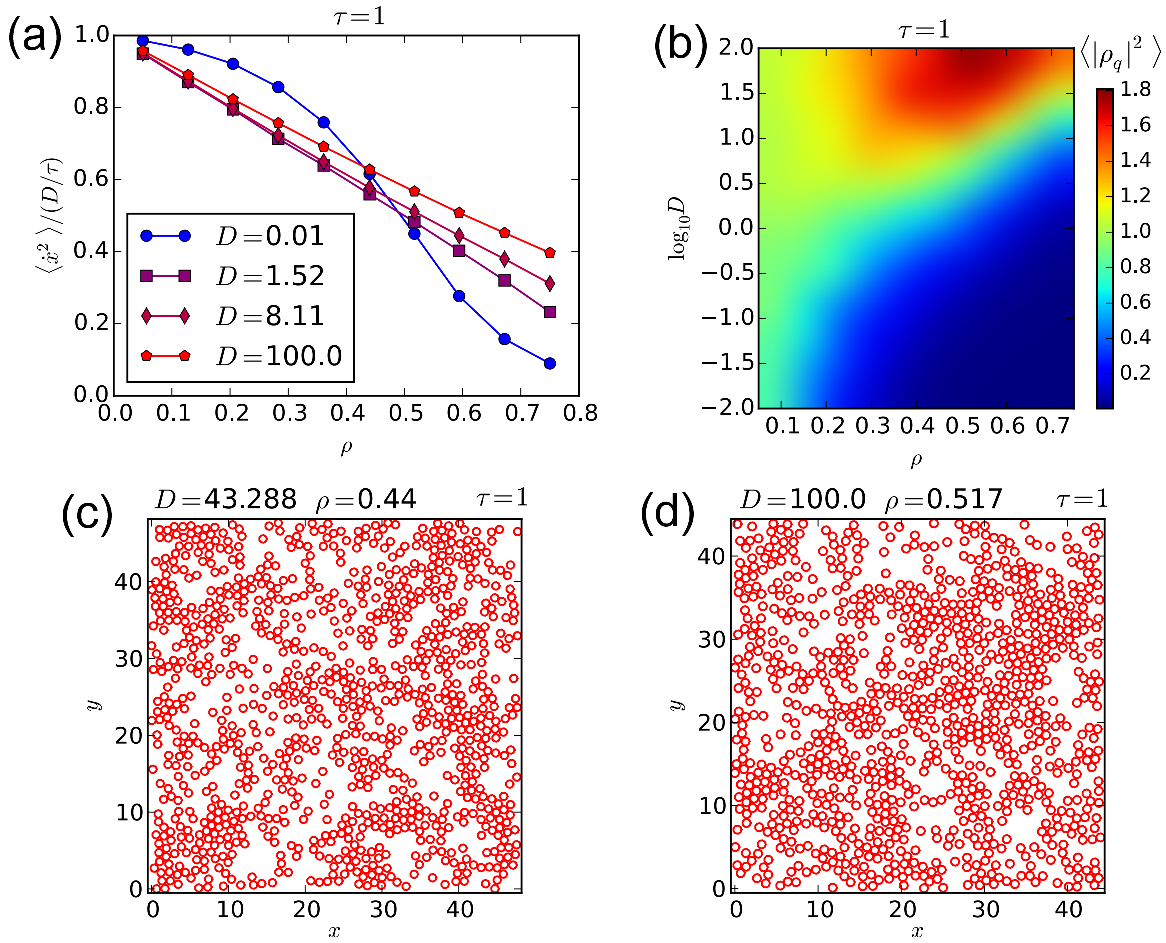

Figure 1: (a) Velocity variance as a function of density at different values of D. (b) The colormap represents the values of the structure factor at low q for several values of the density and the diffusivity. (c) and (d) Snapshot of the system at different values of D and ρ, an evident clustering is observed.

[1] C. Maggi, U. M. B. Marconi, N. Gnan, and R. Di Leonardo, Scientific Reports **5** 10742 (2015).
[2] U. M. B. Marconi and C. Maggi, Soft matter **11** 8768-8781 (2015).
[3] H. Risken, *Fokker-Planck Equation* (Springer, 1984).

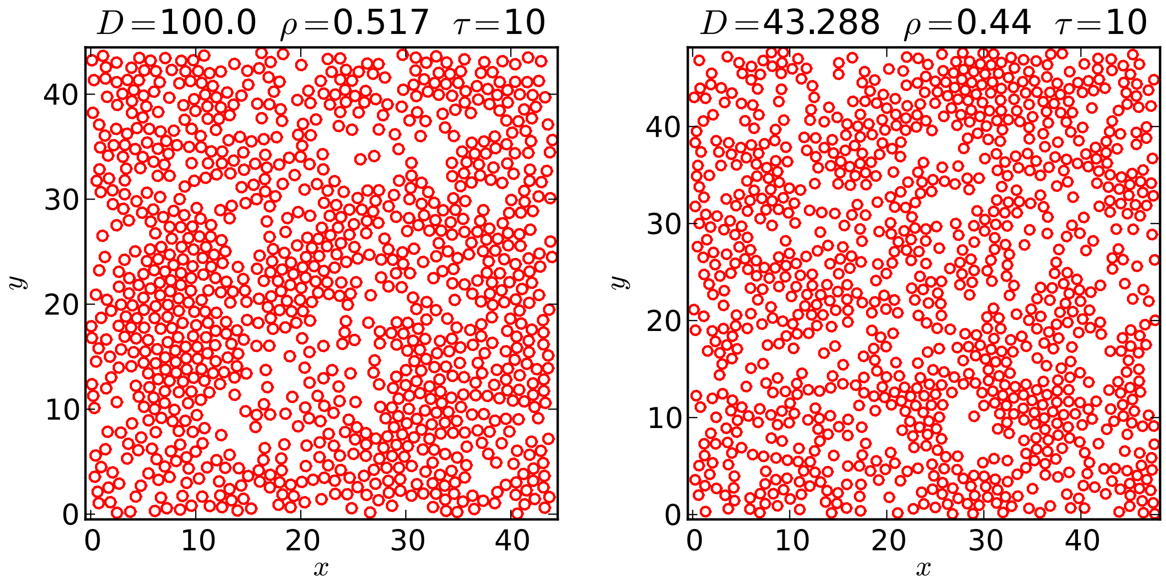

Figure 2: Snapshot of the system at high τ for two different values of D and ρ, an evident clustering is observed without a full phase separation.
